# Intermediate-Salinity Systems at High Altitudes in the Peruvian Andes Unveil a High Diversity and Abundance of Bacteria and Viruses

**DOI:** 10.3390/genes10110891

**Published:** 2019-11-05

**Authors:** Hugo Gildardo Castelán-Sánchez, Paola Elorrieta, Pedro Romoacca, Arturo Liñan-Torres, José Luis Sierra, Ingrid Vera, Ramón Alberto Batista-García, Silvia Tenorio-Salgado, Gabriel Lizama-Uc, Ernesto Pérez-Rueda, María Antonieta Quispe-Ricalde, Sonia Dávila-Ramos

**Affiliations:** 1 Centro de Investigación en Dinámica Celular, Instituto de Investigación en Ciencias Básicas y Aplicadas, Universidad Autónoma del Estado de Morelos, Cuernavaca, Morelos C.P. 62209, Mexico; 2 Departamento de Biología, Facultad de Ciencias, Universidad Nacional de San Antonio Abad del Cusco, Cusco C.P. 0800, Peru; 3 Departamento de Farmacia y Bioquímica, Facultad de Ciencias de la Salud, Universidad Nacional de San Antonio Abad del Cusco, Cusco C.P. 0800, Peru; 4 Escuela de Postgrado, Universidad Nacional de San Antonio Abad del Cusco, Cusco C.P. 0800, Peru; 5 Tecnológico Nacional de México, Instituto Tecnológico de Mérida, Mérida, Yucatán C.P. 97000, Mexico; 6 Instituto de Investigaciones en Matemáticas Aplicadas y en Sistemas, Sede Mérida, Universidad Nacional Autónoma de México, Unidad Académica de Ciencias y Tecnología, Mérida, Yucatán C.P. 97302, Mexico; 7 Centro de Genómica y Bioinformática, Facultad de Ciencias, Universidad Mayor, Providencia, Santiago C.P. 7500000, Chile

**Keywords:** Peruvian Andes, metagenomics, intermediate salinity, microbiome, virus

## Abstract

Intermediate-salinity environments are distributed around the world. Here, we present a snapshot characterization of two Peruvian thalassohaline environments at high altitude, Maras and Acos, which provide an excellent opportunity to increase our understanding of these ecosystems. The main goal of this study was to assess the structure and functional diversity of the communities of microorganisms in an intermediate-salinity environment, and we used a metagenomic shotgun approach for this analysis. These Andean hypersaline systems exhibited high bacterial diversity and abundance of the phyla *Proteobacteria*, *Bacteroidetes*, *Balneolaeota*, and *Actinobacteria*; in contrast, *Archaea* from the phyla *Euryarchaeota*, *Thaumarchaeota*, and *Crenarchaeota* were identified in low abundance. Acos harbored a more diverse prokaryotic community and a higher number of unique species compared with Maras. In addition, we obtained the draft genomes of two bacteria, *Halomonas elongata* and *Idiomarina loihiensis*, as well as the viral genomes of *Enterobacteria* lambda-like phage and *Halomonas elongata*-like phage and 27 partial novel viral halophilic genomes. The functional metagenome annotation showed a high abundance of sequences associated with detoxification, DNA repair, cell wall and capsule formation, and nucleotide metabolism; sequences for these functions were overexpressed mainly in bacteria and also in some archaea and viruses. Thus, their metabolic profiles afford a decrease in oxidative stress as well as the assimilation of nitrogen, a critical energy source for survival. Our work represents the first microbial characterization of a community structure in samples collected from Peruvian hypersaline systems.

## 1. Introduction

Millions of years ago (80–110 million years), the ocean covered the central region of Peru; during the formation of the Andes mountains, these marine waters remained inland and, by evaporation, formed deposits of salt in ponds. Different hypersaline water systems are distributed throughout Peru, such as the salterns of the Acos system and the brines from Maras, two thalassohaline environments located in the Andes mountains in southeast Peru. These two systems have not received much study. Acos is located in the district of Acomayo (southeast Peru) at an altitude of 2852 m above sea level, while Maras is located in the district of Urubamba at an altitude of 3380 m and is composed of 3000 small shallow ponds that form terraces on the slope of the mountain Qaqawiñay (a Quechua word meaning eternal rock) [1,2].

The hypersaline ecosystems are characterized by alkalinity and low oxygen concentrations [3,4,5,6]. Hypersaline aquatic environments are classified into two main categories: (1) thalassohaline environments, which result from the evaporation of seawater and contain a high concentration of NaCl, neutral or slightly alkaline pH, and a salinity exceeding that of seawater by a factor of 5–10; and (2) athalassohaline environments, which are not derived from seawater and contain high concentrations of ions such as Mg^2+^ or Ca^2+^ and a slightly acidic pH [3,4,5,6]. 

Aquatic hypersaline systems represent excellent models for the study of the ecology and diversity of microorganisms. Most saline systems are composed of ponds with different salinity gradients [7]. Microorganisms identified in hypersaline environments have been classified according to the concentration of salts in the environments they inhabit: weak halophiles (1–3% NaCl), moderate halophiles (3–15% NaCl), and extreme halophiles (more than 15% NaCl) [8]. In contrast, there is no generalized classification for saline environments, but they can be divided into low salinity (less than 10% NaCl), intermediate salinity (10–20% NaCl) [9], and high salinity (higher than 20% NaCl) [10].

Regarding microbial communities that live in these ecosystems, a great diversity of microorganisms has been reported, in particular of the *Halobacteriaceae* family within the Archaea domain. For bacteria, the *Halorhodospira*, *Salinibacter*, *Halomonas*, *Chromohalobacter*, and *Salicola* genera are abundant; and eukaryotic organisms such as *Artemia salina*, *Colpodella edax*, and *Dunaliella salina* have been identified in low proportions [5,11,12,13]. In addition, a high diversity of haloviruses has been identified, at concentrations of ≥1 × 10^7^ per mL in seawater, among which a few are cultivable [12].

In this work, the diversity of halophilic microorganisms and functional diversity were determined in two thalassohaline environments, Acos and Maras, that have physicochemical differences in salinity and pH. We expected that these intermediate-salinity environments would contain a greater microbial diversity than high-salinity environments and with a particular microbial community structure given the high altitude. Thus, we consider that this analysis opens diverse opportunities to describe the microbial diversity and functional profile within the Peruvian hypersaline systems and will contribute to knowledge in these environments. This is the first characterization of a microbial community structure of intermediate salinity in samples collected from Peruvian high-altitude salterns.

## 2. Materials and Methods 

### 2.1. Sampling, DNA Extraction, and Sequencing 

Water (20 liters) was collected during the rainy season (January 2018) from two points where the water emerges in the mountain in two hypersaline systems located in Cusco, Peru. The first is in Maras (13°57′59.3″ S, 71°05′65″ W), and the second is in Acos (11°16′25″ S, 72°9′15″ W). The samples were obtained with sterilized tools and containers, and salinity and pH were measured in situ using a hand refractometer (Spectronic Instruments Inc., Rochester, NY, USA) and pH potentiometer (HANNA Instruments, Portugal), respectively. All samples were transported to the laboratory under refrigerated conditions, where liters of water were filtered through 0.22-μm Millipore filters. The DNA was purified from the filters by using ZymoBIOMICS DNA kits (MoBio, West Carlsbad, CA, USA). The DNA concentration was determined using a NanoDrop 1000 spectrophotometer (Thermo Scientific), and fluorometry was measured using a Qubit 4 fluorometer (Invitrogen). The DNA was sequenced using the Illumina NextSeq 500 platform with the Nextera V2.0 kit (150 bp, 2 × 75 bases) at the Instituto de Biotecnología of Universidad Nacional Autónoma de México.

### 2.2. Quality Control and Assembly

The quality control of sequences was performed by FASTQC v0.11.4 software [14], and duplicated sequences were removed using CD-HIT-DUP v4.7 [15] with a maximum mismatch number of 0.03. Reads were assembled in contigs using MEGAHIT v1.1.2 [16] under default parameters in paired-end mode, and contigs of a minimum length of 1000 bp were considered for further analysis.

### 2.3. Microbial Community Taxonomic Assignments

Taxonomic assignments were performed with software Kaiju v16.0. In addition, we used MetaGenome Rapid Annotation Subsystems Technology (MG-RAST v4.03) [17], which compares the assembly sequences with a comprehensive non-redundant database sourced from the National Center for Biotechnology Information (NCBI) databases, and SEED, which categorizes gene function into five levels of resolution. An expected value (E) cutoff of 10^−5^ was employed for taxonomic classifications. Raw data of Metagenomes have been deposited in MG-RAST with accession numbers: mgm4810306.3, mgm4808260.3, and mgm4810472.3.

For virus classification, the viral contigs were achieved with VirSorter v2 [18], and these contigs were classified with MEGAN v5.10.6. For fungi classification, the sequences were compared against a constructed database comprised of 35,296 complete and draft fungi genomes from NCBI. For both viruses and fungi, the best-scoring BLAST results with an E-value of 10^−6^ were parsed, and the taxonomic assignment was determined using MEGAN software [19]. The lowest common ancestor (LCA) method in MEGAN was used for taxonomic assignment, with the following parameters: minimum support of 2; minimum score of 50; top percent of 10.

### 2.4. Diversity Index 

The taxonomic profiles at the species level were used to calculate the diversity indices from all data, and different alpha diversity descriptors were obtained using the Phyloseq function in R v3.3.3 [20]. The beta diversity was determined by Bray-Curtis dissimilarity, and the sampling effort was evaluated through the rarefaction curves using a Vegan library implemented in R [21].

### 2.5. Genome Reconstruction

The reconstruction of the bacterial genome was directed to those species that had the highest abundance according to the taxonomic classification. The genomes were retrieved using the strategy fragment recruitments within Bowtie2 v2.2.6 [22]. The coverage was evaluated using BBmap v38.25 [23], and the consensus sequence was inferred using UGENE v1.31.1 [24]. For the reconstructed genome, the presence of contamination was evaluated using One Codex [25] and Genome Peek. Briefly, One Codex assigns an unknown nucleotide sequence for the identification of k-mers of fixed size k-31 in comparison with its own database. Genome Peek extracts the 16S gene and *radA*/*recA*, *rpoB*, and *groEL*, the principal molecular markers, from a genome for taxonomic identification. The annotation was achieved using Prokka v1.12 [26] and visualized with Genome Atlas. 

For viral sequences, identification was achieved by VirSorter [18] and was based on viral hallmark genes annotated as “major capsid protein,” “portal,” “terminase large subunit,” “spike,” “tail,” “virion formation”, and “coat,” among others. The entire contig was considered viral if more than 80% of predicted genes on a contig had a viral signal. This software finds new viruses at different confidence levels, with scores of categories 1 to 4, with 4 being the highest confidence level. Viral sequences identified within category 1 by VirSorter were visualized with the easyfig v2.2.2 tool and also assessed with the PHAge search tool (PHAST) [27].

Finally, contigs with lengths of ≥10 kbp within category 2 (“quite sure”) in VirSorter were translated into protein sequences and classified taxonomically using the vConTACT v2 software [28] with default parameters (https://bitbucket.org/MAVERICLab/vcontact), with the aim of classifying these possible new viruses.

### 2.6. Binning for Putative Genomes

Assembled contigs were clustered into bins or metagenome-assembled genomes (MAGs), using MaxBin v2.2.4 [29]. Briefly, MaxBin performs genome reconstruction from metagenomes based on two genomic characteristics, tetranucleotide frequencies and the level of bin coverage, using single-copy marker genes. The two metagenomes from Acos were used to recover the MAGs, which were later annotated with Prokka [26]. 

From the annotation of MAGs, the ribosomal sequences were extracted in single copy (L2, L3, L4, L5, L6, L14, L15, L16, L18, L22, L24, S3, S8, S10, S17, and S19), and then these sequences were aligned with those reported by Hung et al. [30] by using MAFFT v7.005 for taxonomic identification [31]. The phylogenetic analysis was performed using FastTree v2.1.7 [32], which considers an approximate maximum likelihood with 100 bootstrap replicates. Finally, the phylogenetic tree was displayed using ITOL [33].

### 2.7. Functional Analysis and Biogeochemical Cycles 

Prodigal v2.6.3 [34] was used for predicting protein-coding genes in the assembled contigs by using the metagenomic mode, and the functional assignment was achieved using SUPERFOCUS [35], which contains the SEED database with an E-value of 10^−5^. From functional abundance tables, a heatmap using the ggplot2 library [36] and RColorBrewer library in R (www.ColorBrewer.org) was generated. Finally, microbial metabolic pathways involved in the biogeochemical cycles for carbon, sulfur, nitrogen, hydrogen, iron, and oxygen were identified using the Multigenomic Entropy-Based Score pipeline (MEBS v1) with a false-discovery rate of 0.0001 [37].

## 3. Results and Discussion

### 3.1. Site Characterization and Field Sampling

**Figure 1:**
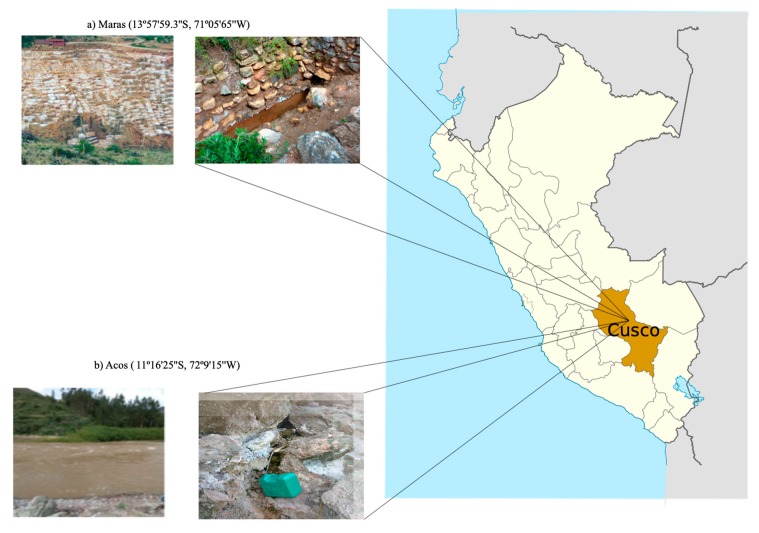
Location of the field sites in Cusco Peru. (**a**) Maras with 3000 shallow ponds. (**b**) Acos at the origin of the water.

The water samples were collected from two locations in the district of Cusco, Peru. The first sample was collected from Maras; its pH was 7 and its salinity concentration was 23% NaCl (Figure 1). This concentration was slightly lower than previously reported (25% NaCl) in emergent water, whereas in the crystallizer ponds the concentration was higher (30% NaCl) [1].

The second and third samples were collected from Acos, with a pH of 7.9 and 19% salinity (Table 1). The salinities of the thalossohaline water samples from Maras and Acos [1,2] were similar to levels in other solar salterns with intermediate salinity, such as Marine Saltern in Santa Pola, Spain (13–19% NaCl) [7,38] and Saltern in Isla Cristina, Spain (21% NaCl) [38,39]. In this regard, salterns exhibiting an intermediate salinity have been found to contain a greater diversity of microorganisms than salterns with higher salt concentration [38]; the concentration of NaCl defines the diversity and structure of the microbiome in these environments [40].

### 3.2. Community Structures of Intermediate Hypersaline Systems 

In order to analyze the diversity, abundance, and genes involved in metabolic profiles of samples from Maras and Acos, shotgun metagenomic sequencing was performed. Maras and Acos salterns can be considered environments at high altitude with intermediate salinity (according to the determined percentage of salt) (Table 1). However, salinity is not the only parameter that modifies the abundance and diversity of microorganisms present in these ecosystems; biogeographic patterns that may also have a role include altitude, remoteness of these environments, oxygen availability, alkalinity, altitude, and UV irradiation [41,42,43].

With the metagenomes obtained from the two locations, the general structure of the microbiome was determined. To this end, the sequences were classified with Kaiju (Table 1), and the results showed a high abundance of bacterial organisms (~57% of the sequences), followed by Archaea (~16%). These results contrast with the abundance reported in crystallizer ponds in Maras, where the salinity of >30% NaCl showed a microbiota dominated by Archaea (80–86% of total counts) with much lower percentages of Bacteria (10–13%) [1].

The enrichment analysis of species and diversity in these sites, evaluated with Chao, Shannon, and Simpson indexes, revealed that Acos samples had a greater richness than Maras samples (Supplementary Table S1). These results correlated with the rarefaction curves, i.e., in Acos samples, the asymptotic distribution was reached, which indicates a greater diversity showing correlation to the other diversity indexes, whereas in the Maras sample the asymptote was not reached, since most of the contigs were assigned to *Cutibacterium acnes*, which is highly unlikely to reside in this environment and was considered a contaminant and was therefore eliminated from diversity curves and subsequent analyses. However, the remaining organisms present in this sample are halophilic, but as shown in the diversity curve it is necessary to perform new sampling to know the diversity in Maras (Supplementary Figure S1). In addition, the Bray-Curtis dissimilarity index was performed to evaluate the beta diversity, showing an index equal to 1, which indicates a different species composition between Maras and Acos. In contrast, the index value between the two samples from Acos was close to zero, suggesting that these samples contained the same species (Supplementary Figure S2).

These results correlate with findings reported for other saltern ponds with intermediate salinity, such as those in Santa Pola, Spain, with 13–19% NaCl, where high abundance levels of bacteria (~73 and ~54%) and archaeal organisms (~27% and ~46%, respectively) were found [38,44]. The same was found when the Chao index was compared for these metagenomes [45]. In contrast, in the saltern pond located in Isla Cristina, Spain (21% NaCl), Archaea were predominant (~84%), followed by Bacteria (~16%) [38]; although the structure at the phylum level is equivalent, important differences at the genus level are attributed to particular local ecological conditions [38]. 

These results suggest that in environments with higher salt concentrations there is less diversity and species richness, probably because there is lower availability of nutrients and oxygen, in contrast to intermediate-salinity environments, where there is a greater availability of nutrients and oxygen. Therefore, salt concentration is an important factor that shapes the structure of the microbial community in hypersaline environments and determines its diversity and abundance.

### 3.3. Bacterial and Archaeal Community Composition

**Figure 2:**
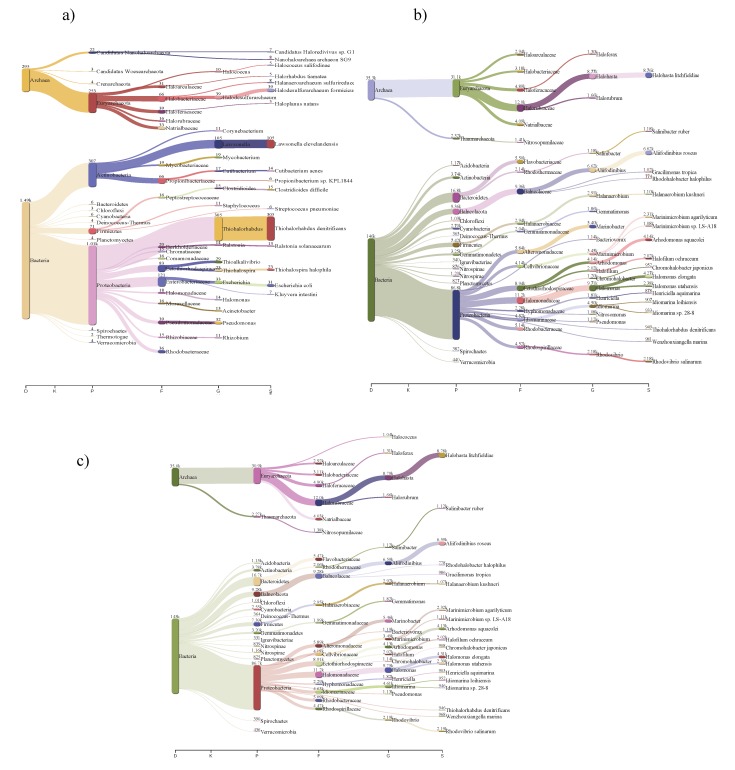
Taxonomic profile in hypersaline metagenomes from Cusco, Perú. (**a**) Maras; (**b**) Acos 1; (**c**) Acos 2. On the *x*-axis are the taxonomic levels: D, domain; P, phylum; C, class; O, order; F, family; G, genus; S, species. Numbers correspond to the assigned contigs.

Previous studies have shown that the halophilic world is highly diverse, but this diversity is reduced with increasing salt concentrations [46]. In the case of intermediate-salinity environments, several moderately halophilic bacteria have been reported, including *Halomonas*, *Salinivibrio*, *Halobacillus*, *Thalassobacillus*, *Bacillus*, *Salinicoccus*, *Idiomarina*, *Chromohalobacter*, and *Salinicoccus* [7,38,47,48,49]. In the metagenomic samples from Maras, bacteria from the phylum *Proteobacteria* (38%) were the most abundant, followed by *Actinobacteria* (11.58%), *Firmicutes* (2.68%), *Cyanobacteria* (0.40%), *Bacteroidetes* (0.40%), *Deinococcus-Thermus* (0.26%), and *Verrucomicrobia* (0.26%) (Figure 2).

At the species level in Maras salterns, it was interesting that the most abundant bacterium was *Thiohalorhabdus denitrificans* (11.51%), which is an extremely halophilic species [50], followed by *Thiohalospira halophila* (0.87%) [51]. Both of these species are chemolithoautotrophic sulfur-oxidizing bacteria which use thiosulfate as the electron donor [50,51], and neither has been reported previously in intermediate-salinity settings.

Other halophilic bacteria, such as *Pseudomonas* (2.15%) and *Halomonas* (0.94%), were identified in lower proportions than previously reported [1,44,47]. Even the main bacteria described in hypersaline systems, such as *Salinibacter ruber* [52,53] and *Rhodovibrio salinarum* [1], were found in low abundance (~0.07%, each species) in our study, probably because the altitudes of these sites affect bacterial structures, as we have shown.

In addition, predominant non-halophilic bacteria found included *Lawsonella clevelandensis* (7.06%), *Escherichia coli* (2.08%), *Clostridium difficile* (1%), *Cutibacterium acnes* (0.9%), and *Ralstonia solanacearum* (0.8%). The presence of non-halophilic bacteria in hypersaline environments has been previously described in the Santa Pola saltern (19% NaCl), and some of these organisms have developed adaptation mechanisms, such as a strong GC bias, as has been identified in halophilic organisms as a strategy to avoid UV-induced thymidine dimer formation [44,45,54,55].

The two samples from Acos exhibited similar compositions of microorganisms: *Proteobacteria* corresponded to ~59% of identified sequences, followed by *Bacteroidetes* (11%)*, Balneolaeota* (6%)*, Firmicutes* (5%), and *Actinobacteria* (2%). Both Acos metagenomes had the same composition as environments of intermediate salinity previously reported, showing a high diversity and abundance of bacteria [7,38]. Interestingly, in Acos salterns members of the *Balneolaeota* phylum were identified, including moderate halophiles (5–10% NaCl) abundant in sediments, saline soils, and marine habitats [55,56] (Figure 2b,c).

At the level of genus, *Halomonas* was the most abundant (8.4%), with more than 70 different species identified in Acos; *Halomonas elongata* (2.8%) was the most abundant, followed by *Halomonas utehensis* (1.6%). In this regard, organisms of the *Halomonas* genus are aerobic heterotrophic, halo-alkaliphilic, and sulfur-oxidizing bacteria and are commonly found in intermediate-salinity, high-altitude environments [57,58]; they are also a source for the production of bioplastic polyhydroxyalkanoates [59].

In contrast, at the species level, the most abundant bacteria were *Aliifodinibius roseus* (~5%) within the phylum *Balneolaeota*; this species is considered moderately halophilic (6–10% NaCl for optimal growth). Also abundant were two species, *Halomonas elongata* (2.93%) and *Arhodomonas aquaeolei* (2.84%), an obligately halophilic bacterium with optimal growth at 15% NaCl; both of these species have been shown to degrade phenol [60]. To our knowledge, only a few reports have described these bacteria in a metagenome from an intermediate-salinity environment. *Marinimicrobium agarilyticum*, *Rhodovibrio salinarum* (1.50%), *Salinibacter ruber* (0.80%), and *Idiomarina* sp. (0.64%) were in low abundance. *Idiomarina loihiensis* is a bacterium identified in environments a wide range of temperatures (from 4 °C to 46 °C) and salinities (from 5% to 21%) that presents polyextremophile behavior [61].

In both the Maras and Acos sites, the low abundance of *S. ruber* is understandable, since this bacterium prefers environments with higher salinity.

Therefore, different species of moderately halophilic bacteria were found in Acos, with *Proteobacteria* the most abundant. These results correlate with findings from another high-altitude saltern located in Atacama, Chile, at 2,700 m above sea level, where halophilic bacteria able to grow at intermediate salinity were isolated [62]. In general, the moderately halophilic bacteria are aerobic or facultative anaerobic microorganisms that belong to different genera, as part of a physiologically heterogeneous group of bacteria [47]. 

In intermediate-salinity salterns, such as the Peruvian hypersaline systems, the abundance of archaeal organisms is low, as found in the Maras samples, where *Euryarchaeota* organisms were found to be highly abundant, followed by “*Candidatus* Nanohaloarchaeota,” and “*Candidatus* Woesearchaeota.” In both samples from Acos, *Euryarchaeota* organisms were the most abundant, followed by *Thaumarchaeota*, *Crenarchaeota*, and “*Candidatus* Bathyarchaeota.” Within the *Euryarchaeota* phylum, the *Halobacterium* family was found to be predominant, similar to findings from other salterns and salty lakes [6,45,63,64].

In Maras, *Halodesulfurarchaeum formicicum* was the most abundant species. *Halodesulfurarchaeum* is a novel anaerobic genus that was discovered in a deep-sea salt-saturated anoxic environment and in sediments from hypersaline lakes [65].

In Acos, the most abundant archaeon was *Halohasta litchfieldiae* (~3.5%), a chemoorganotrophic aerobic that can grow in salt concentrations around 12–28%, presenting adaptation to low temperatures [66,67,68] as occurs in the area of the Peruvian Andes where minimum temperatures reach between –7 °C and –4.4 °C.

**Figure 3:**
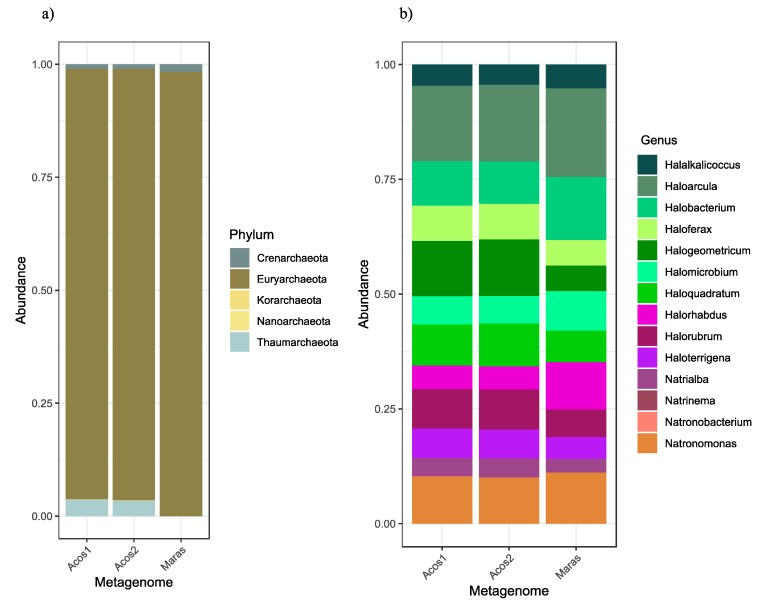
Taxonomic classification of archaea according to MetaGenome Rapid Annotation Subsystems Technology (MG-RAST). (**a**) Composition of the archaeal community at the phylum level, where *Euryarchaeota* prevail. (**b**) Diversity within the class *Halobacteria.* The genus *Haloarcula* prevails in all samples.

The taxonomic assignment analysis was also carried out with MG-RAST; the abundance of archaea was low, in accordance with the results of Kaiju. From the class *Halobacteria*, 14 different genera were identified, with *Haloarcula* genus the most predominant in the three samples (Figure 3). The presence of this genus is interesting because it has been reported to be involved in recombination processes. This process could be occurring between bacteria and archaea, among their sharing genes, for example, rhodopsin family genes which are common and have different functions such ion pumps, channels, enzymes, photosensory receptors that could favor the adaptation [69,70].

### 3.4. Composition of the Viral Community

Although viruses are sources of genetic variation, as they can modify a genome’s plasticity and alter the structure of populations and also biogeochemical cycles, few reports have described the structure of virus communities in hypersaline environments [71,72,73,74]. In this work, the taxonomic assignment performed with Kaiju revealed that 0.2% to 1.32% of the reads were associated with viruses (Table 1). This was probably because we did not perform a viral enrichment with our samples; however, it was possible to find viruses, because they would be included within the host cells or in the form of proviruses [75]. 

Because of the low percentage of detected viruses in the samples, we used Virsorter, which detects the viral signal in metagenomic datasets [18]. From the assembly of reads, we identified the viral contigs according to VirSorter, and they were subsequently classified taxonomically with MEGAN (See Materials and Methods).

The results identified the order *Caudovirales*, specifically, the *Siphoviridae*, *Podoviridae*, and *Myoviridae* families, in the samples; indeed, these families seem to be ubiquitous in marine environments [76]. 

**Figure 4:**
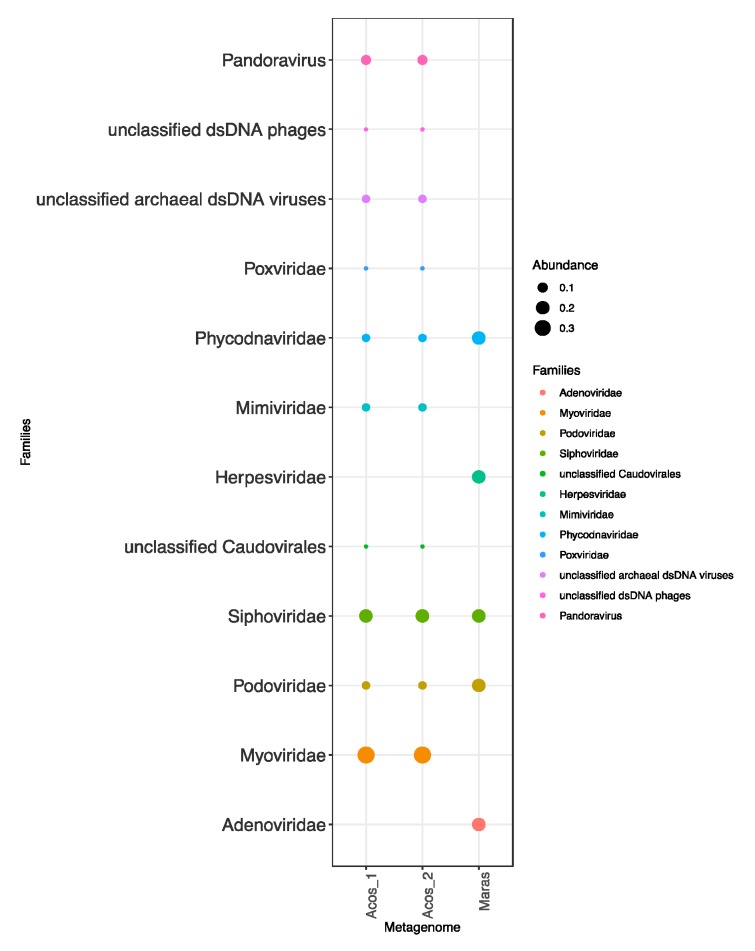
Compositions of the viral communities at the species level in Acos and Maras.

Interestingly, in Maras eukaryotic viruses such as *Adenovirus* and *Herpesvirus* were identified, probably as a consequence of the composition of eukaryotic organisms in the samples, as also reported for Red Sea brines [77]. Additional double-stranded DNA (dsDNA) viruses associated with eukaryotes were also found in the Acos samples, mainly viruses from the *Phycodnaviridae, Poxviridae*, *Mimiviridae*, and *Pandoravidae* families (Figure 4); all of these are Megavirales, which are nucleocytoplasmic large DNA virus (NCLDVs). NCLDVs infect animals and unicellular eukaryotes [78] found in other hypersaline environments, such as the Salton Sea in the United States and Organic Lake in the Antarctic [79]. 

Another important group of viruses found in Acos was an unclassified archaeal dsDNA virus (Figure 4); this virus has been reported in high abundance in hypersaline environments, with spindle-shaped morphologies of *Haloarchaea* viruses, but this happens when salt concentration reaches saturation, where in general *Archaea* are predominant [12,72,80].

In summary, we identified six virus families associated with eukaryotic cells and five families that infect *Bacteria* and *Archaea*. This last group was the most abundant, according to the microbial composition in this environment.

### 3.5. Composition of Fungal Communities

**Figure 5:**
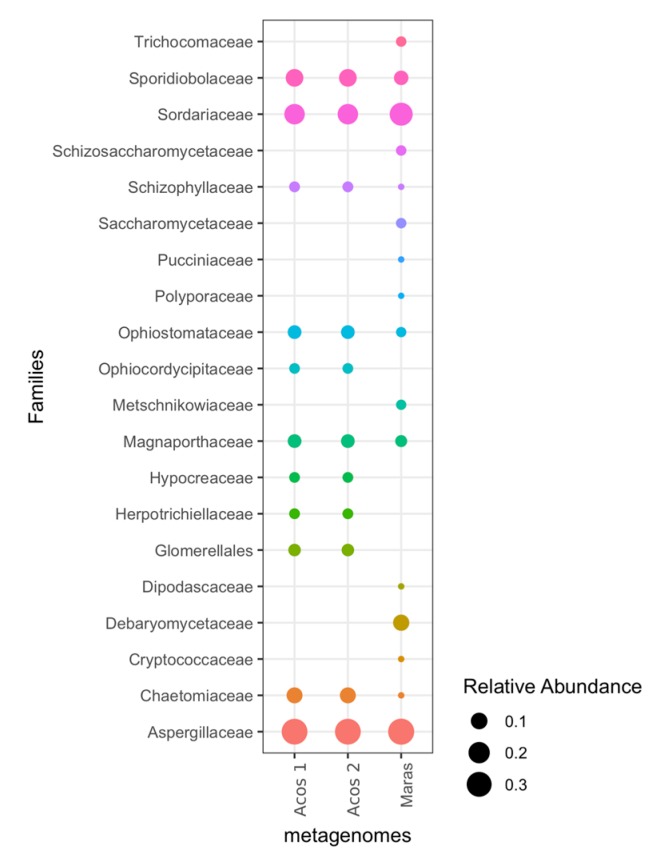
Diversity taxonomy of fungi. The *Aspergillaceae* family was the most abundant in Acos and Maras.

The diversity of microorganisms in intermediate-salinity systems is not restricted to prokaryotes. Approximately 2% of the reads corresponded to eukaryotes. According to the Megan classification system, two phyla of fungi were found, *Ascomycota* (with 85%) and *Basidiomycota* (with 10%), as has been reported for other hypersaline environments [81]. At the family level, the most abundant were *Arpergillacea*, followed by *Sordariaceae*, *Sporidiobolaceae*, and *Chaetomiaceae* (Figure 5). *Aspergillus* has been reported to be dominant in salterns of Slovenia, along with *Cladosporium* and *Penicillium* [82]. These filamentous fungi are ubiquitous and have been isolated with high frequency in hypersaline environments [83]. Some species in the *Sordariaceae* family have also been isolated from hypersaline environments. The *Sporidiobolaceae* family is within the *Basidiomycota* phylum, which has been recovered from sea water, glacier ice, and extremophile environments. *Rhodotorula* was recovered from hypersaline ponds in Israel [84]. The *Chaetomiaceae* family was recovered together with 19 inhabiting hyphomycetes fungi in soils from the hypersaline Urmia Lake [85]. Thus, a high diversity of fungi has been found in hypersaline environments, where the most abundant are melanized *Aspergillus*, which is a ubiquitous genus used in biotechnology applications for its production of citric acid and enzymes, and non-melanized *Rhodotorula*, which comprises several species that can be used in bioremediation [86].

In summary, the results show an important diversity of fungi within the hypersaline environments; however, their functions are still unclear.

### 3.6. Genome Reconstruction

#### 3.6.1. Bacterial Genome Reconstruction in A Hypersaline Environment

One of the aims in this work was the reconstruction of complete genomes that could provide information about the main metabolic pathway associated with hypersaline metabolism. To this end, the genomes were retrieved using a fragment recruitment strategy with the reads aligned against the available reference genome [87], and their integrity was assessed with Genome Peek [88] and One codex [25] (see Materials and Methods).

According to the abundance levels found with our taxonomic assignments, the most abundant genomes were *Halomonas elongate*-like and *Idiomarina loihiensis*-like and these were retrieved from the metagenome. The complete reference genomes reported in NCBI for these bacteria were used for fragment recruitment.

**Figure 6:**
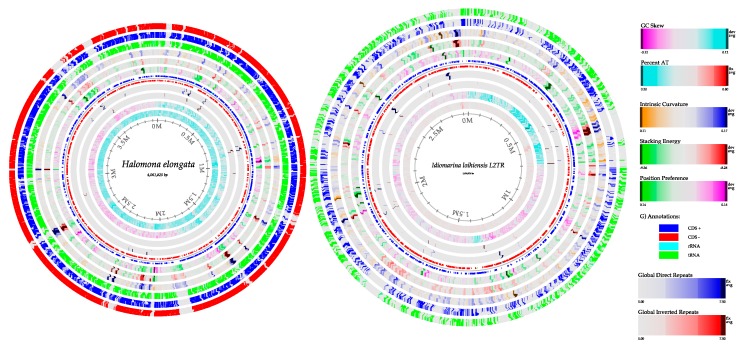
Draft genome comparison of *Halomonas elongata* and *Idiomarine loihiensis*. From the outside to center of the genomes of *Halomonas elongate*-like from Acos 2 (red) and Acos 1 (blue), the innermost rings show percent AT, GC skew, GC content, global inverted repeats, CDS-, CDS+, position preference, stacking energy, and intrinsic curvature, which are colored in a gradient, and the interpretation of the references to color is in the figure legend. The *Idiomarine loihiensis*-like genome from Acos 2 (green) and Acos 1 (blue) and the innermost rings show the same interpretation of genome *H. elongata*.

First, the *Halomonas elongate*-like genome was reconstructed, comparing the sequences against the reference genome MAJD01000001.1. Both samples of Acos showed 93% breadth coverage and a deep coverage of 8.3x The circular chromosome of ~3.7 Mb is graphically represented in Figure 6; it has a GC content of 64%, similar to *Halomonas elongata* isolated from Huanoquite at Peru. The average nucleotide identities (ANIs) between the reference genome (MAJD01000001.1) and the genomes from Acos 1 and Acos 2 were 98.04% and 98.02%, respectively (Table 2).

A comparison between *Halomonas elongata* strain HEK1 (MAJD01000001.1), *H. elongata* strain MH25661 (QJUB00000000.1), and the two genomes recovered from this work revealed that all strains share a core of 2,984 genes. Indeed, both genomes recovered from Acos share 916 genes, a significant number of genes in comparison to the other genomes. In addition, *Idiomarina loihiensis*-like, reported as a predominant genome in a saline environment, was also assembled. The coverage was 74–78% with a reference genome (*I. loihiensis L2TR* GCA_000008465.1), and the deep coverage was 5-6x for Acos 1 and Acos 2. Both strains share 93.0% of identity according to their ANIs. Finally, the two genomes share 2151 common genes (Supplementary Figure S3).

Interestingly, the reconstructed genomes of *Halomonas elongata* and *Idiomarina loihiensis* show different strategies for maintaining osmotic equilibrium, according to the annotation; de novo synthesis of the ectoine pathway is complete in *H. elongata*. Ectoine is a compatible solute of low molecular weight of aspartate metabolism, which is produced when there are increased K^+^-glutamate levels [89]. In contrast, in *Idiomarina loihiensis*, de novo synthesis of ectoine was absent; however, we identified genes encoding ABC transporters such as the ATP-dependent Na+ exporter *natAB*, in addition to other iron transporters, which promote detoxification in hypersaline environments. These findings show different adaptation strategies of bacteria in hypersaline environments. 

The annotation of the genes exclusively shared between two *Halomonas* genomes from Acos revealed that most of them were related to nitrogen metabolism, chemical reactions, and pathways involving organic acids. Regarding the genes related to the metabolism of nitrogen, genes encoding a nitrate/nitrite sensor protein, nitrate reductase, and ammonia monooxygenase were found. This is interesting since *Halomonas* use nitrogen as the last acceptor of electrons even in conditions of low oxygen, as is the case in hypersaline environments [90].

In general, these *Proteobacteria* play an important role in the nitrogen cycle, through recycling of nitrogen by assimilation of gaseous nitrogen from the atmosphere and decomposition of organic matter, causing nitrogen to be constantly available [90].

#### 3.6.2. Reconstruction of Viral Genomes

**Figure 7:**
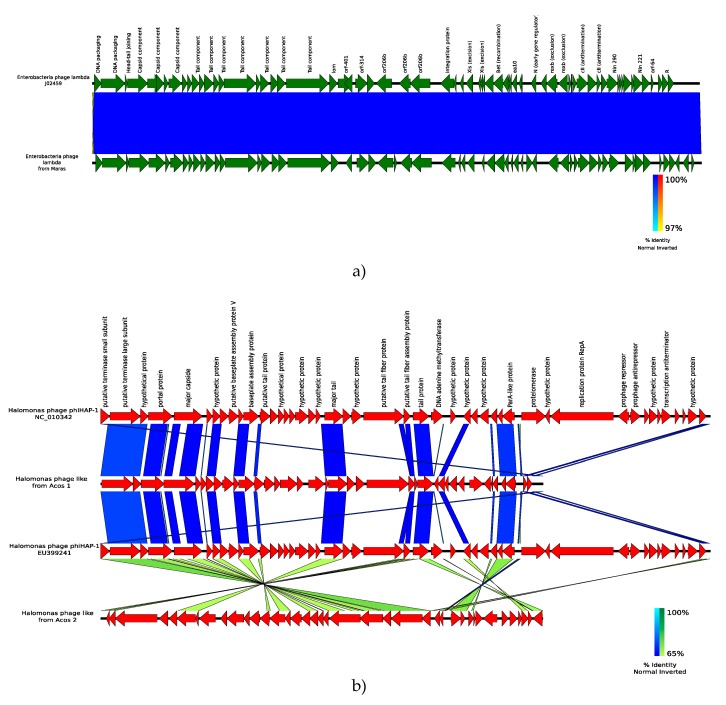
Genomes of novel bacteriophages. (**a**) *Enterobacteria* phage lamda-like from Maras; (**b**) *Halomonas* phage-like (phiHAP-1) from Acos.

Traditional techniques limited us in obtaining viral genomes, but through metagenomics it was possible to reconstruct these genomes, allowing us to expand knowledge about the influence of viruses in this particular environment. In this regard, the viral contigs identified correspond to bacteriophages, as expected, since bacteria were more abundant in our metagenomes. In the Maras sample, a genome with 97% similarity with the lambda phage of *Enterobacteria* (*Siphoviridae* family) was found. This phage infects *Escherichia coli*, a non-halophilic bacterium that was abundant in this sample (Figure 7a).

In Acos samples, around 100 different contigs with viral signals were identified; because many of these could be fragments of viral sequences, different criteria were used, such as the presence of inverted terminal repeats in the case of circular genomes and similarities in size lengths with a reference genome (no more than 10% of size length) [18].

In the Acos samples, two phages of *Halomonas elongata* were recovered. This finding was somewhat expected, since *H. elongata* is abundant in these intermediate-salinity environments, but this is the first time that bacteriophages have been reported in this bacterium. The two recovered phages have a size length of approximately 28 Kbp, and a comparative analysis with two ΦHAP-1 reference genomes revealed that they have the same pattern of synteny and a protein identity greater than 65%. (Figure 7b).

The phage used for comparison was *Halomonas* phage ΦHAP-1. This is a *Hapunavirus* belonging to the family *Myoviridae* and was isolated from *Halomonas aquamarina*. The GC content in ΦHAP-1 is 59%, which is slightly lower than other phages such as ΦHAP-1, found in Acos with a 64% GC content, and similar to the GC content of the host genome (*H. elongata*) [91].

The ΦHAP-1-type phages from Acos have 40 putative open reading frames (ORFs) with 6 genes fewer than the reference genome. Genes coding for proteins such as the RepA replication protein, the prophage repressor, the prophage antirepressor, and the protelomerase were not identified; the latter is necessary for the maintenance of the linear state of the prophage within the host genome [92]. In addition, inverted repeats were found at positions 28,428–28,452 to 28,455–28,479 with a length size of 25 bp and an identity of 100%, suggesting that the genome is in a circular form, because this kind of inverted repeated sequence is usually found in regions processed by protelomerases and originates from the release of phage with covalently closed ends. All of these findings suggest that the phage could be in their free form.

**Figure 8:**
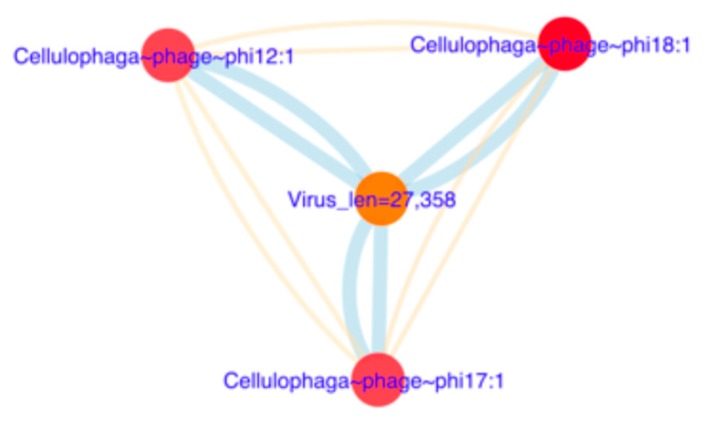
Protein-sharing network of genome of *Cellulophaga* phage. Yellow lines indicate strong similitarity, and blue lines indicate weak similarity. Thus, the virus of length 27,358 bp could be a novel virus.

The rest of the viral sequences obtained in the metagenomes through BLAST analysis had very poor identity with sequences in the NCBI database, and they were used for taxonomic or functional allocation. Thus, the contigs of >10 kb was clustered using the Viral RefSeq database and vConTACT2; this tool allows classification of viral sequences with protein comparisons. In Figure 8, two examples of viral assignation taxonomy are presented. In Figure 8 a viral sequence with ~27 Kpbs shares identity with proteins from *Cellulophaga* phage, which infects algae typically found in marine environments. In Figure 8 are four viruses with size lengths of about ~11 to 30 Kpbs that shared identities with different enterophages, showing a mosaicism as a reflection of horizontal gene transfer. In total, 27 sequences could have a taxonomic assignment as new viruses with this strategy (Supplementary Figure S4). 

#### 3.6.3. MAGs 

**Figure 9:**
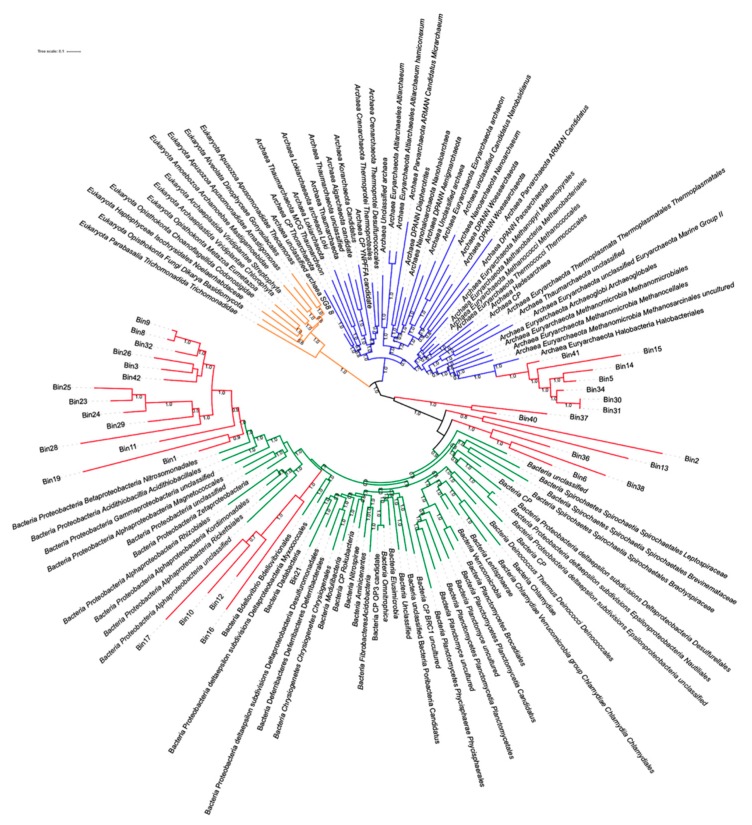
Phylogenetic tree, including the binned sequences associated with different taxa clades. Branches in orange correspond to Eukaryota; branches in blue correspond to Archaea; branches in green correspond to Bacteria; Branches in red correspond to bins. Bootstrap levels are noted.

Another strategy to retrieve new genomes from metagenomic sequences with little or no identity with sequences already reported is by binning, in which genomes are assembled without a reference sequence. The binning method has the aim to classify contig sequences in a specific taxon, called metagenome-assembled genomes (MAGs). The binning methods can also describe novel species in these environments. The binning of metagenomic sequences was performed only for Acos, because at least two samples with the same origin are necessary to enrich the data. From this, a total of 42 bins were assembled in annotated draft genomes, and their ribosomal genes were extracted. However, some of this process resulted in a low degree of completeness. Therefore, we performed a phylogenetic analysis that revealed that 31 MAGs were classified within a specific domain and seven of them were closely related to the *Halobacteria* class (Bin 5, Bin 14, Bin 15, Bin 31, Bin 30, Bin 34, and Bin 41) (Figure 9) within the *Euryarchaeota* phylum, which is predominant in hypersaline environments.

Four MAGs were closely related with *Alphaproteobacteria* unclassified (Bin 10, Bin 12, Bin 16, Bin 17), and 14 MAGs were closely related to *Gammaproteobacteria.* Indeed, most of the found bacteria corresponded to *Proteobacteria*, in accordance with the bacteria found in our metagenomes (Bin 1, Bin 3, Bin 8, Bin 9, Bin 11, Bin 19, Bin 23, Bin 24, Bin 25, Bin 26, Bin 28, Bin 29, Bin32, Bin 42). Therefore, in this analysis the *Proteobacteria* phylum prevails over the *Euryarchaeota* phylum in Acos samples, indicating that salinity plays an important role in the structure of the community of microorganisms that inhabits this ecosystem.

The strategies for the reconstruction of genomes, such as fragment recruitment and MaxBin, offered different results, since the first strategy is a targeted search and for the second strategy the search starts from scratch in obtaining draft genomes. Another important difference is that the latter assemblies, coming from more than one metagenome, could build chimeric genomes. However, with the two strategies, genomes of *Gammaproteobacteria* similar to *Halomonas* could be reconstructed.

### 3.7. Functional Community Composition

The strategies that halophilic organisms use to survive in hypersaline environments are diverse and include thickening of the cell wall, increase in pigmentation, production of compatible solutes, solute transport mechanisms, and production of antibiotic proteins to limit the growth of other populations [93]. Therefore, we analyzed the functional composition of microorganisms in intermediate-salinity environments in order to determine how these mechanisms are potentially used by microorganisms in these environments.

**Figure 10:**
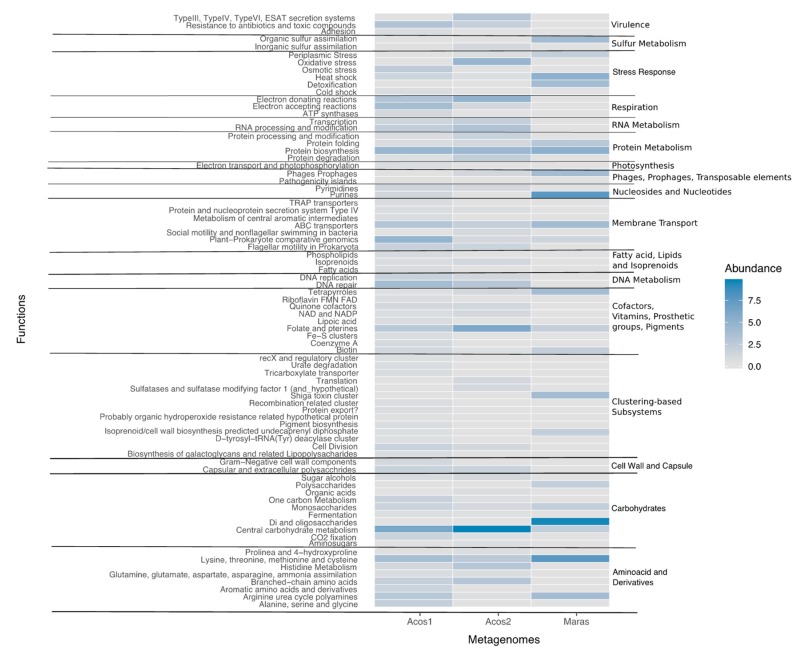
Heatmap of the relative abundance of proteins based on SEED classifications.

Thus, the contigs from hypersaline metagenomes were annotated using SEED subsystems, and these results revealed that 11–13% of coding sequences from Acos and 14% of those from Maras were related to metabolism of carbohydrates (central carbohydrate metabolism, synthesis of monosaccharides and polysaccharides) (Figure 10).

The genes classified into the category of amino acids and derivatives functions were present in ~8% to 12% in Acos and ~11% in Maras. Overall, in the three metagenomes the synthesis of lysine, threonine, methionine, and cysteine were the more abundant categories. This correlated with the fact that in some halophilic bacteria there is a preferential use of codons to encode these amino acids [94]. In this regard, most of these amino acids are hydrophobic, found on the inside of proteins, especially in hypersaline environments, which strengthen the hydrophobic interactions [92].

Other categories overrepresented, with ~6.7% to ~9.77% abundance in samples, were respiration, functions related to donating/accepting electrons, and ATP synthases. All of these participate in the transfer of electrons to obtain energy.

Interestingly, the category related to pigment functions was found in 8% to 10% Acos sequences and 7% of Maras sequences. The class *Halobacteriaceae* is mainly responsible for α-bacterioruberin pigment, a pink-red product in hypersaline environments. In addition, *Salinibacter ruber* is responsible for producing salinixanthin carotenoid, a C-40 acyl glycoside carotenoid that also contributes to the coloration of salterns. This bacteria and these pigments are important in hypersaline environments as they reduce the UV irradiation that damages DNA, which tends to be high in these saline environments [9]. 

The category of membrane transport was present in ~3% to ~5% abundance; in particular, the membrane proteins in Gram-negative bacteria were more abundant than in Gram-positive bacteria, including the YrbG Na^+^/Ca^2+^ cation antiporter, a very important protein in this kind of saline environment. This system has been reported in *Haloarchaea*, which have a wide variety of ion transporters, to have a role in regulating fluctuating salinity levels and avoiding osmotic shock [95]. In other salterns with intermediate salinity, such as Santa Pola (13% NaCl), genes related to this function have been reported to be overrepresented [45]. 

The function of resistance to antibiotics and toxic compounds was also found to be abundant in these metagenomes, including pathways involved in sulfur heavy metal cycling, cobalt–zinc–cadmium resistance, and also copper homeostasis and resistance to arsenic. Since heavy metals such as arsenic do not have biological roles, low concentrations are toxic to the cell, and therefore microorganisms have mechanisms for reduction. Many Archaea have different heavy metal transporters [96].

In addition, the stress category was more abundant in Maras (~14%) than in Acos (~6%). This category includes predominant functions such as oxidative stress, osmotic stress, heat stress, detoxification, cold shock, and periplasmic stress. These types of functions were prevalent in Maras, and although the concentration of salt was higher in Maras than in Acos, a greater presence of non-halophilic organisms was identified in Maras. It is well known that organisms growing in high concentrations of salt accumulate stress molecules, such as reactive oxygen species, and the organisms must therefore have mechanisms for their detoxification [97]. 

In the same way, samples from Acos presented abundant oxidative stress functions. Reactive oxygen species in hypersaline environments are common, thus organisms in these environments have detoxification mechanisms. In particular, in microaerophilic and anaerobic metagenomes, oxygen-detoxifying enzymes have been identified, such as superoxide dismutases, catalases, peroxidases, and glutathione peroxidase [98]. In the Acos metagenome, we identified enzymes involved in the response to oxidative stress, such as 5-oxoprolinase, and enzymes responsible for maintaining the reducing environment, such as glutathione reductase, glutathione hydrolase (involved in reduction of glutathione disulfide), and hydroperoxide resistance (responsible for detoxification of organic hydroperoxides).

Since the microorganisms are under oxidative stress, it is common to identify redundant enzymes responsible for DNA repair [99]. However, reactive oxygen species are not the only compounds that modify the genetic material; other agents that produce data in the genetic material include UV light exposure and desiccation, and so, as expected, the functions of DNA synthesis and DNA repair are the most represented in proteins found in the Acos samples. 

According to our analysis with MG-RAST, detoxification enzymes were identified in Archaea, within the classification “housecleaning nucleoside triphosphate pyrophosphatases”; all of these belonged to class Nudix hydrolases, including nucleoside 5-triphosphatase and 5-nucleotidase SurE. In Bacteria, these enzymes were found in high abundance, as was the dimeric dUTPase enzyme. Interestingly, viruses also possess detoxification enzymes of this category, in particular the enzyme deoxyuridine 5′-triphosphate nucleotidohydrolase, that decreases the intracellular concentration of dUTP so that uracil cannot be incorporated into viral progeny DNA. 

All of the above enzymes are responsible for the elimination of damaged nucleotides caused by reactive oxygen species. For viruses, the incorporation of damaged nucleotides in nucleic acids is detrimental to replication of viral progeny. In this way, the virus could contribute to the adaptation of the host to its environment. 

Regarding DNA repair, we found bacterial systems that contribute to this function, among which were base excision repair (BER), repair of DNA double-strand breaks (DSBs) (RecBCD pathway and RecFOR pathway), nucleotide excision repair (NER), and DNA mismatch repair (MutL-MutS system). However, the mechanisms of nucleotide excision repair (NER) and DNA mismatch repair (MutL-MutS system) were more abundant in Bacteria. The function related to nucleotide excision repair has also been reported to be overrepresented in hypersaline environments [100].

In addition, we identified eight proteins related to DNA DSB repair in the annotations for viral sequences; this is one of the most common damaging events [101]. However, bacteriophages and some NCLDV possess homologous proteins, such as Rad50/SbcC, which is probably involved in the processing of dsDNA ends for processing during recombination [102]. These proteins were also identified in circular genomes of bacteriophages, such as *Vibrio parahaemolyticus* bacteriophage [103], which could indicate that these proteins are also propagated in this type of virus and could have implications in the repair of genetic material in stress environments. Other genes for methyltransferase enzymes, which are ubiquitous in the prokaryotic world and are associated with host protection of DNA damage, were also identified in our viral sequences.

Other functionally important genes found in viral sequences were auxiliary metabolic genes (AMGs) originally from the genome host. The AMGs found were ribonucleotide reductases and *phoH*, among others. The ribonucleotide reductases are associated with lytic rather than temperate viruses, and the *phoH* gene plays a role in the transport of phosphate in conditions of starvation. *Synechococcus* and *Prochlorococcus* (cyanophages) carry AMGs; however, in this study we found these families were in low abundance, as they are predominantly found in marine environments, so it correlates with the abundance of these families reported above [104].

### 3.8. Metabolic Pathway Involved in Biogeochemical Cycles 

**Figure 11:**
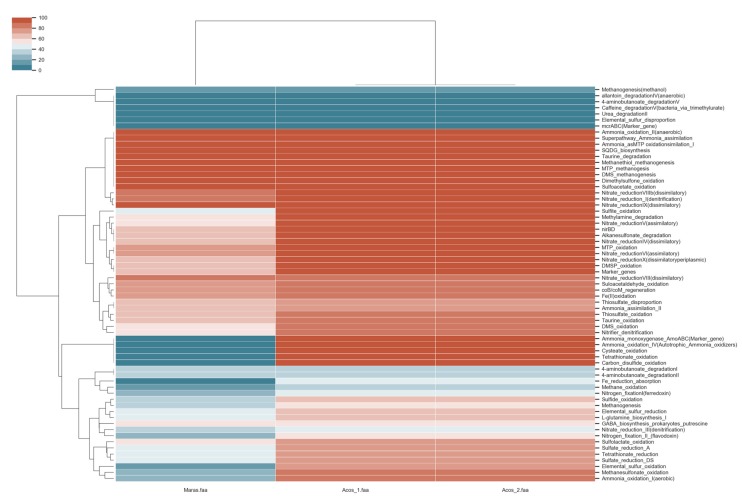
Completeness pathways of biogeochemical cycles. Mainly the pathways of nitrogen and sulfur cycles are complete within the hypersaline samples.

In order to evaluate the contribution of different metabolic pathways in the biogeochemical cycles associated the metagenomes, MEBS software was used to analyze the three samples. From this analysis, only two complete pathways of the carbon cycle were identified (Figure 11), while the nitrogen and sulfur cycles in the samples were more highly represented (Figure 11). In the case of nitrogen, the pathways of denitrification and the reduction of nitrate by assimilation were found to be more prevalent, since that nitrite is generally produced under anoxic conditions such as in hypersaline environments [6]. On the other hand, the reduction of dissimilatory nitrate (nitrite-ammonia) involving the proteins encoded by the genes *nirB*, *nirD*, *nrfA*, and *nrfH* is generally more highly expressed in *Proteobacteria*, *Bacteroidetes*, *Euryarchaeota*, and *Verrucomicrobia* [90]. Those were found as complete in our metagenomes, which correlates with the great abundance of *Proteobacteria* in the metagenomes.

Because oxygen is limited, denitrification (nitrate-nitrite) is another pathway that contributes to the nitrogen cycle. In addition, species in the environment use nitrogen as a source of growth [105,106]. These pathways were also found as complete in the metagenomes of Acos, which indicates the importance of nitrogen in hypersaline environments. In this pathway, the proteins encoded by the genes *narGHIJ*, *napAB*, *nirKS*, *norBC,* and *nosZ* are included; these genes are expressed by *Bacteroidetes*, *Euryarchaeota,* and *Proteobacteria.* In addition, the *narL* gene in the virus compensates for the metabolic pathways of the microorganisms for nitrogen metabolism [90,107]. Finally, in the Maras sample, partially complete denitrification pathways were found (a 40-60% of representation), indicating that microorganisms can contribute to the reduction of nitrate and nitrite for the production of N_2_.

Some organisms, such as *Proteobacteria* and *Thaumarchaeota*, are responsible for producing nitrate by nitrification at high salt concentrations [93,108], as well as the route of nitrogen fixation; however, they were partially complete, despite nitrite being an important energy source (Figure 11). In this regard, nitrite is not the only source of energy in this environment, since many Archaea and some bacteria use sulfur compounds as donors or electron acceptors for energy production [109]. In this case, the pathways related to sulfite oxidation, oxidation of sulfur DMS, and oxidation of dimethylsulfoniopropionate (DMSP) were found to be complete. Mainly, DMSP has been reported in abundance, which indicates that DMSP is an important source of carbon and energy [110]. Therefore, Bacteria and Archaea contribute to the oxidation of DMSP as an energy source, at different proportions.

## 4. Conclusions

In this study, we present a snapshot of microbial and functional diversity of two intermediate hypersaline environments in the Peruvian Andes, based on a metagenomics shotgun approach. The intermediate salinity environments show a great diversity and abundance of bacteria, more so than the archaea in the samples. At the level of phylum, *Proteobacteria* are the most abundant and predominated over other bacteria and archaea. However, the *Balneolaeota* phylum was found only in Acos in great abundance, but was not diverse. In addition, we reconstructed the draft genomes of *H. elongata* and *I. loihiensis,* which have different mechanisms of adaptation to hypersaline environments, via de novo synthesis of ectoine and *natAB* transporters, respectively. Also, we obtained whole genomes from bacteriophages. Functional analysis indicated that microorganism in hypersaline environments contribute to the biogeochemical cycles involving carbon and nitrogen as the source of energy. We also found genes related to oxidative stress and DNA repair. Interestingly, viruses also had such repair protein genes, which are otherwise exclusive to eukaryotes and bacteria. This study contributes to the current knowledge of intermediate-salinity environments at high altitudes.

## Figures and Tables

**Table 1:** Sequence features of hypersaline metagenomes from Cusco, Peru.

Data Set	Salinity	pH	Number of Paired-End Reads	Number of Contigs Assembled	Sequences		Taxonomical Classification			
					Classified	Unclassified	Bacteria	Archaea	Eukarya	Viruses
Acos 1	19%	7.9	63,387,998	257,314	71%	29%	57%	14%	2%	0.2%
Acos 2	19%	7.9	79,304,621	256,430	71%	29%	57%	16%	2%	0.2%
Maras	23%	7.0	56,086,809	2650	70%	30%	56%	11%	1%	1.32%

**Table 2:** Features of *Halomonas elongata* and *Idiomarina loihiens* is genomes with number of transfer RNA (tRNA), transfer-messenger RNA (tmRNA) and ribosomal RNA (rRNA).

Features	*Halomonas elongata* (Acos 1)	*Halomonas elongata* (Acos 2)	*Idiomarina loihiensis* (Acos 1)	*Idiomarina loihiensis* (Acos 2)
Length size (bp)	3,768,127	3,763,770	2,111,175	2,227,077
% GC	64	64	47.2	47.3
CDS	4564	4678	4060	3871
tRNAs	65	69	55	56
tmRNA	1	1	1	1
rRNAs	12	12	12	13

## Supplementary Materials

The following are available online at https://www.mdpi.com/2073-4425/10/11/891/s1, Figure S1: Rarefaction curves based at level species diversity, Figure S2: Dendrogram of all samples. Analysis of beta-diversity was carried out at species level using hclust and Bray-Curtis dissimilarity, Figure S3: Pangenome of draft genome (a) *Halomonas elongata* (b) *Idiomarina loihienis*, Figure S4: Protein-sharing viral network of virus from samples of Acos, Table S1: Indexes of diversity.
