# Supplementary material for: Intermediate-Salinity Systems at High Altitudes in the Peruvian Andes Unveil a High Diversity and Abundance of Bacteria and Viruses
Source: Genes (Basel). 2019 Nov 5;10(11):891. doi: 10.3390/genes10110891 (PMC6895999; doi:10.3390/genes10110891)
Supplement: Supplementary file 1 [file genes-10-00891-s001.pdf]

Supplementary Table and Figures

Table S1. Indexes of diversity

Sample	Observed	Chao1	Se.Chao1	Shannon	Simpson
Maras	210	271.18	20.05	2.23	0.60
Acos1	425	443.75	9.90	4.26	0.96
Acos2	429	449.31	10.51	4.27	0.96

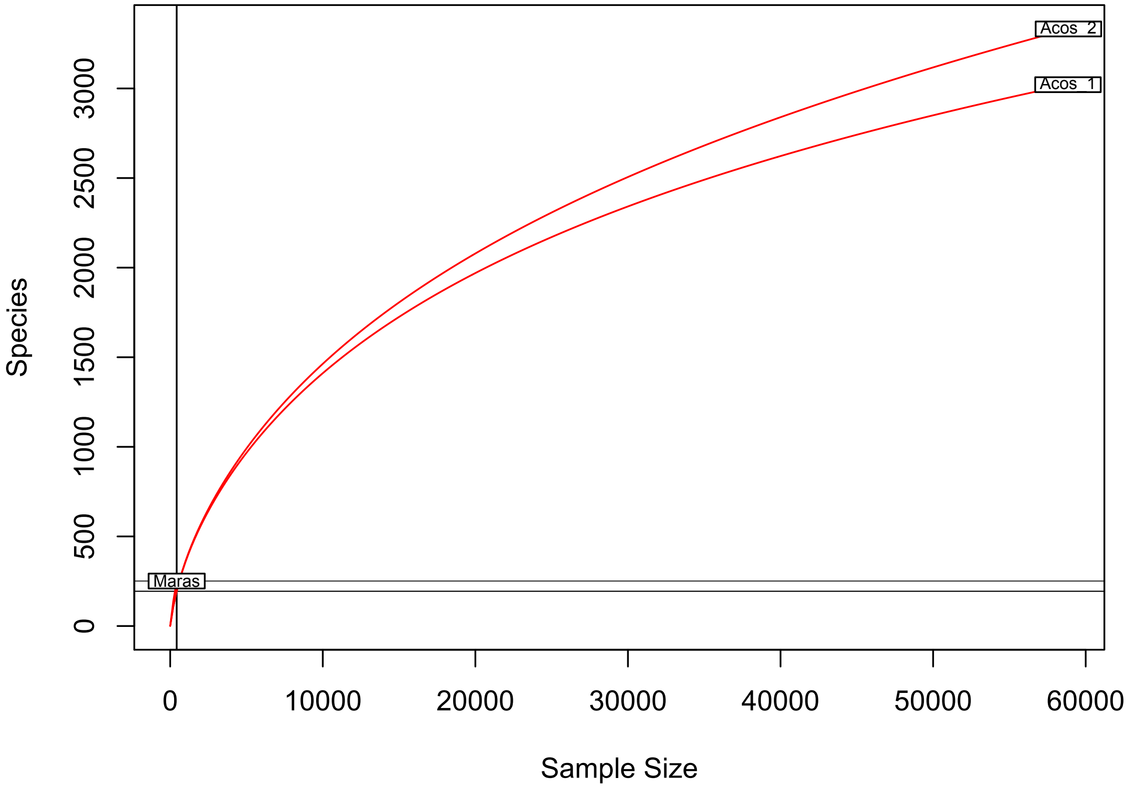

Figure S1. Rarefaction curves based at level species diversity

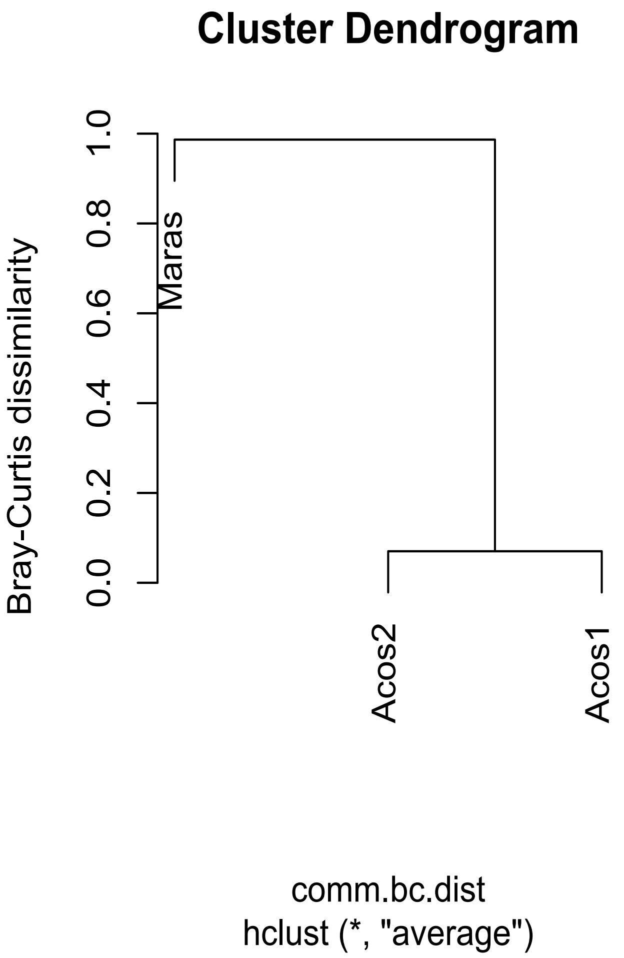

Figure S2. Dendrogram of all samples. Analysis of beta-diversity was carried out at species level using hclust and Bray-Curtis dissimilarity.

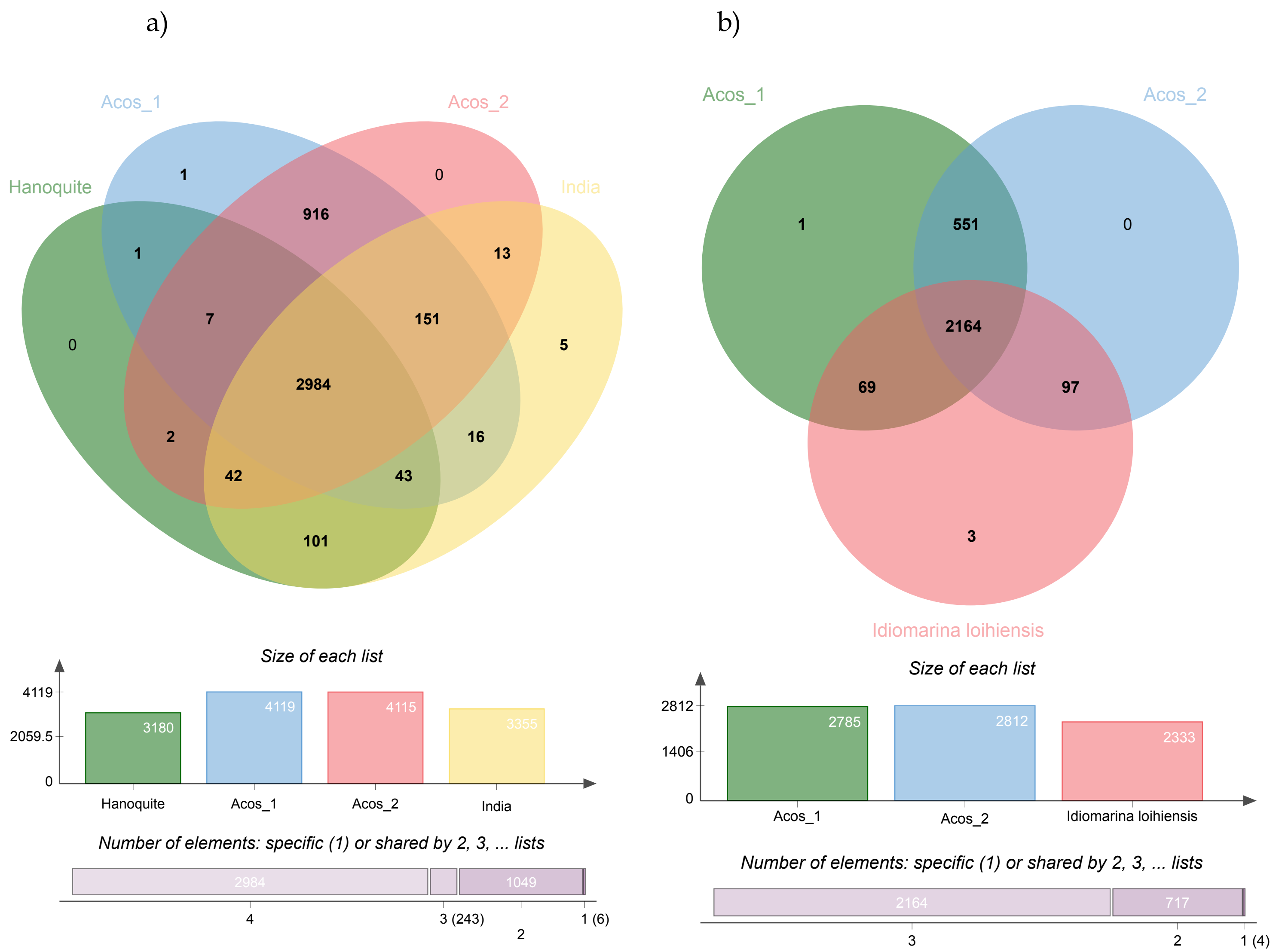

Figure S3. Pangenome of draft genome a)*Halomonas elongata* b) *Idiomarina loihienis*

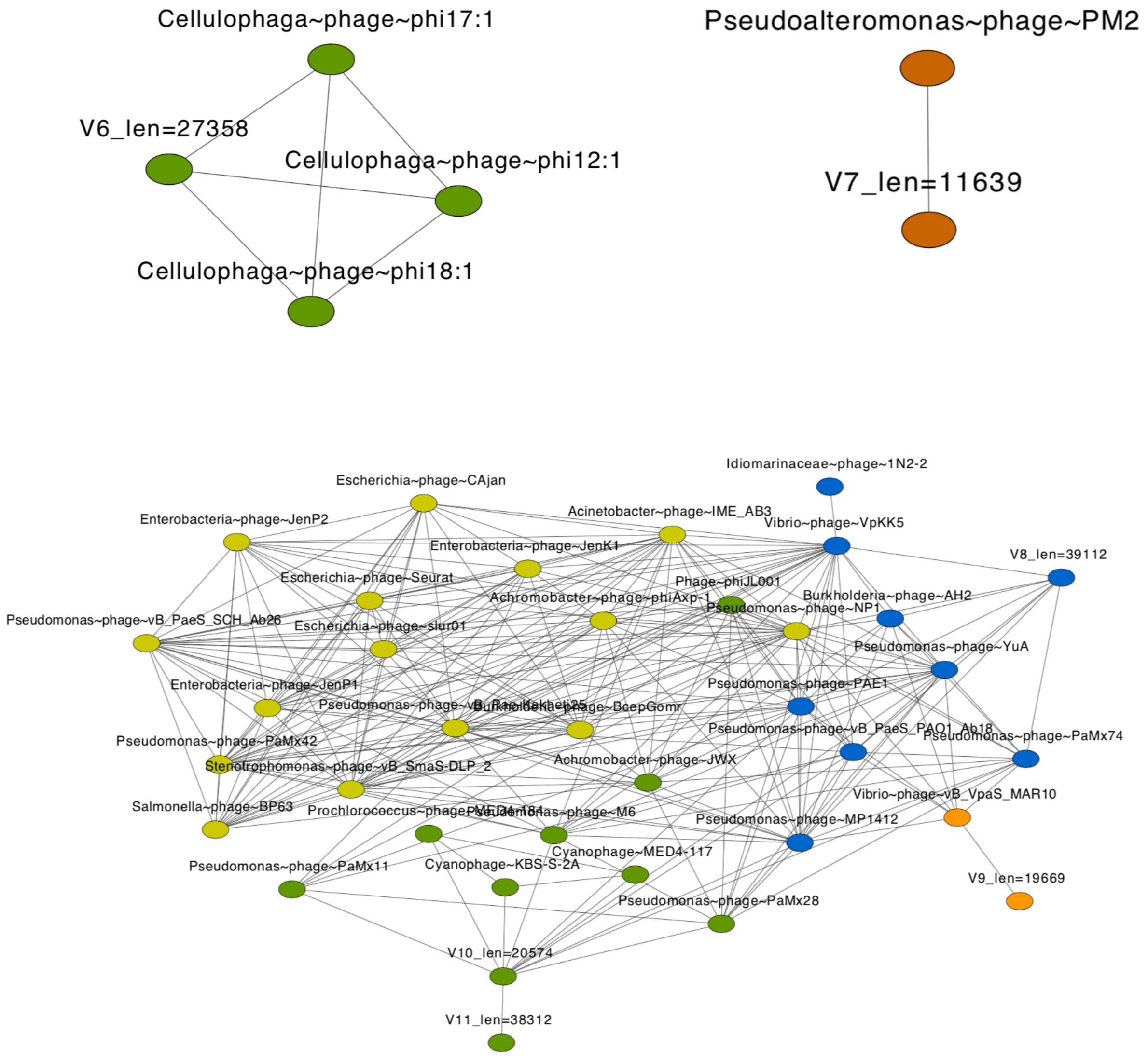

Figure S4. Protein-sharing viral network of virus from samples of Acos.
